# Effects of greenery at different heights in neighbourhood streetscapes on leisure walking: a cross-sectional study using machine learning of streetscape images in Sendai City, Japan

**DOI:** 10.1186/s12942-023-00351-6

**Published:** 2023-11-08

**Authors:** Shusuke Sakamoto, Mana Kogure, Tomoya Hanibuchi, Naoki Nakaya, Atsushi Hozawa, Tomoki Nakaya

**Affiliations:** 1 Graduate School of Environmental Studies, Tohoku University, 468-1 Aoba, Aramaki, Aoba-Ku, Sendai, 980-8572 Japan; 2 The Endowed Department of Traffic and Medical Informatics in Disaster, Tohoku Medical Megabank Organization, Tohoku University, 2-1 Seiryo-Machi, Aoba-Ku, Sendai, 980-8573 Japan; 3 Graduate School of Letters, Kyoto University, Yoshida Honmachi, Sakyo-Ku, Kyoto, 606-8501 Japan; 4 Graduate School of Medicine, Tohoku University, 2-1 Seiryo-Machi, Aoba-Ku, Sendai, 980-8573 Japan; 5 Preventive Medicine and Epidemiology, Tohoku Medical Megabank Organization, Tohoku University, 2-1 Seiryo-Machi, Aoba-ku, Sendai, 980-8573 Japan

**Keywords:** Walking behaviour, Leisure walking, Street greenery, Shade, Google Street View (GSV)

## Abstract

**Background:**

It has been pointed out that eye-level greenery streetscape promotes leisure walking which is known to be a health -positive physical activity. Most previous studies have focused on the total amount of greenery in the eye-level streetscape to investigate its association with walking behaviour. While it is acknowledged that taller trees contribute to greener environments, providing enhanced physical and psychological comfort compared to lawns and shrubs, the examination of streetscape metrics specifically focused on greenery height remains largely unexplored. Therefore, this study examined the relationship between objective indicators of street greenery categorized by height from a pedestrian viewpoint and leisure walking time.

**Methods:**

We created streetscape indices of street greenery using Google Street View Images at 50-m intervals in an urban area in Sendai City, Japan. The indices were classified into four ranges according to the latitude of the virtual hemisphere centred on the viewer. We then investigated their relationship to self-reported leisure walking.

**Results:**

Positive associations were identified between the street greenery in higher positions and leisure walking time, while there was no significant association between the greenery in lower positions.

**Conclusion:**

The findings indicated that streets with rich greenery in high positions may promote residents’ leisure walking, indicating that greenery in higher positions contributes to thermally comfortable and aesthetic streetscapes, thus promoting leisure walking. Increasing the amount of greenery in higher positions may encourage residents to increase the time spent leisure walking.

## Background

Walking for leisure or recreation (hereafter, referred to as leisure walking) as a form of physical activity, has been noted to be important for physical and mental health [1, 2]. While walking for transportation, such as for errands or commuting, has been found to be more prevalent in areas with macroscale walkable environmental attributes, typically high residential density, diverse land use mixed, and high road connectivity, the environmental elements that encourage leisure walking are more ambiguous [3]. Additionally, leisure walking is commonly linked to the perception of micro-scale environmental qualities, such as aesthetic streetscapes [4, 5]. A Japanese study investigating the correlation between walking purposes and environmental perceptions in residential neighbourhoods across four cities highlighted that longer leisure walking times are associated with safer road traffic and improved aesthetic environments. This emphasizes the importance of micro-scale walkable features directly perceived by pedestrians, such as pleasant streetscapes, rather than macro-scale walkable features [6]. Amid the micro-scale walkability components, the inclusion of streetscape greenery, measured through streetscape images, has garnered significant attention in recent years. Research has indicated that individuals residing in neighbourhoods with more streetscape greenery are more inclined to participate in leisure walking and physical activities [7–10].

These findings, which illustrate how street greenery promotes leisure walking, align well with seminal works on urban design arguing that street greenery enhances the pedestrian comfort, the appeal of streetscapes and adds value to residential areas [11]. Traditionally, to comprehensively capture the human eye-level conditions of street greenery over a wide area, researchers have often resorted to questionnaire surveys, interviews, or field surveys conducted by trained auditors. However, each of these methods has faced criticism for potential biases in assessment results, as well as for the considerable time and expenses involved [12]. This limitation is now being mitigated by the increased availability of eye-level streetscape imagery, exemplified by platforms like Google Street View (GSV), Mapillary and Baidu Total View, and advancements in machine learning techniques, particularly semantic segmentation. These advancements automatically extract object measurements of landscape components including greenery from streetscape images over a wide area [12–15]. Indicators derived from such streetscape indicators have been found to closely align with people's real perception of the environment while walking, as demonstrated by Aikoh et al. [16], who established a strong correlation between machine learning-based street greenery assessments and those made by a trained auditor. It is worth noting that detailed geospatial information using aerial photography and LiDAR (Light Detection and Ranging) can also serve as a method of obtaining indicators on street greenery [17, 18]. For example, Tsai et al. [18] reported a positive association between street-level tree cover engagement in active transportation, such as walking and biking. This connection was established using 1-m resolution landcover data generated from both aerial photographs and LiDAR. However, quantification of greenery based on the number of trees or through aerial and satellite images can differ from what is actually perceived by individuals on the ground, particularly in areas with dense vegetation [12, 19].

Many previous studies on street greenery predominantly emphasized the quantity of greenery, primarily examining green visibility in a scene. However, contemporary research increasingly delves into additional dimensions beyond quantity, including the analysis of green quality and variations in vegetation types [20–24]. For instance, in a study conducted in New York City, it was observed that self-reported health improved in areas with higher densities of taller trees in the neighbourhood. Conversely, no discernible correlation was found for areas with lower greenery, such as lawns. This led to the argument that taller trees may offer greater benefits, such as enhanced shade, noise reduction, and psychological restoration, compared to lower greenery [23]. Tabatabaie et al. [24] emphasized the significance of shading, often generated by the canopies of tall trees, in enhancing the thermal comfort and aesthetic appeal of the streetscape. This, in turn, encourages physical activity on the streets. Considering this, discussions on green quality often incorporate aspects such as tree shade and high canopy lines [17, 22]. These elements contribute to the creation of a comfortable environment for pedestrians, facilitating an improved walking experience on the roads. Further, as explained by Appleton's prospect and refuge theory, individuals tend to prefer places where they can “see without being seen”, fostering a sense of safety and coziness [25]. In the realm of architectural design, it has been observed that the presence of tall greenery casting shades can evoke a sense of refuge [26]. These findings indicate that greenery positioned at greater heights may significantly contribute enhancing both the physical and psychological comfort of individuals on streets, thereby encouraging walking activities. However, previous studies examining the association between walking behaviour and greenery in the streetscape have not considered the role of greenery ‘height’ in this context.

This study used eye-level streetscape imagery to determine the specific height of street greenery, as perceived by pedestrians, was most associated with leisure walking time per week. We hypothesised that more greenery at higher positions from the pedestrian's perspective would lead to more walking than greenery at lower positions. Although this approach does not provide an exhaustive assessment of the quality of the greenery, it is expected to contribute to an improved objective assessment of the streetscape greenery from a pedestrian perspective.

## Methods

### Study area

**Fig. 1:**
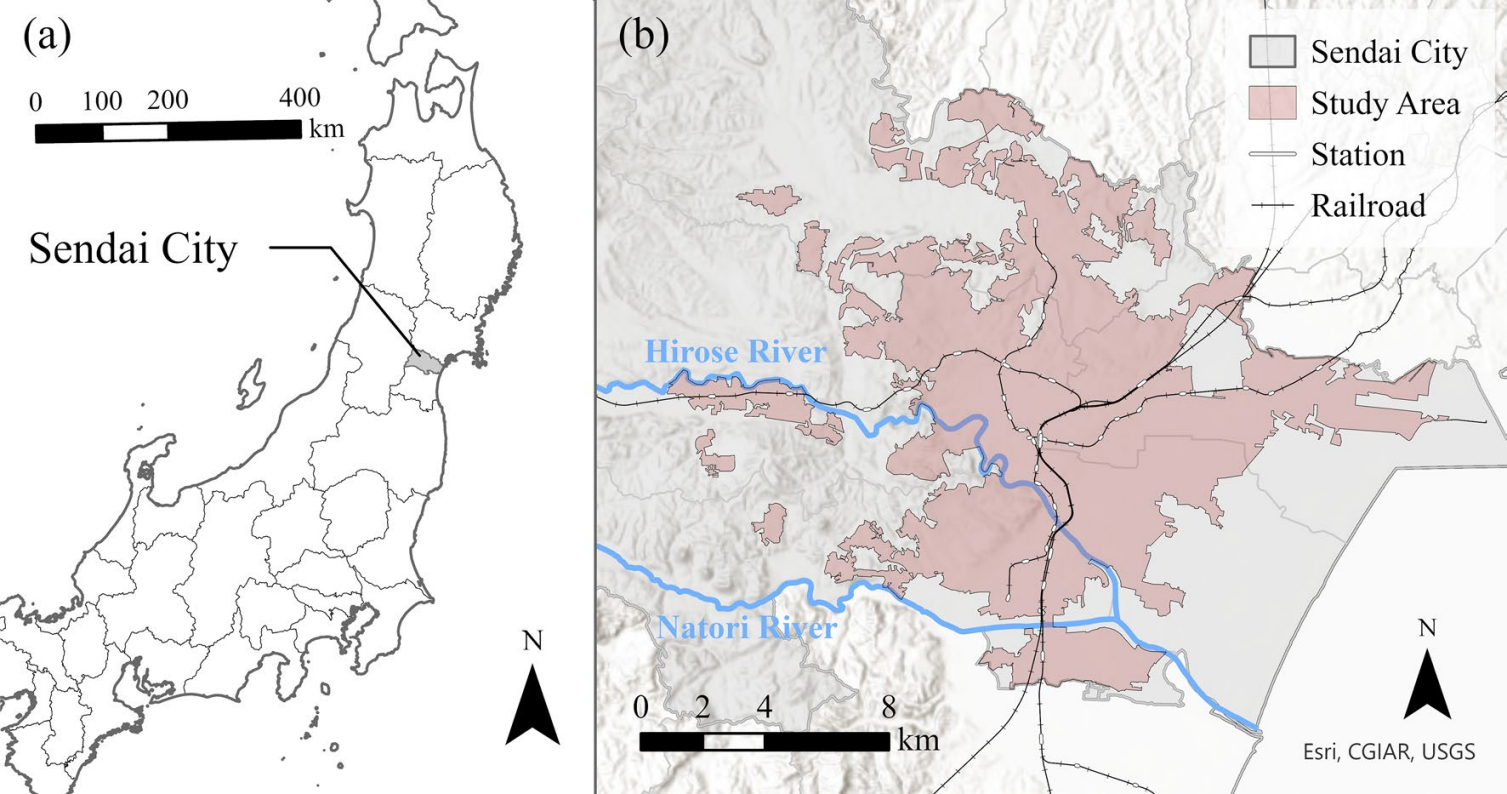
Study area location: **a** the location of Sendai City in Miyagi Prefecture, Japan; **b** the extent of study area in Sendai City

To examine the urban environment, this study focused on participants residing in urban areas, referred to as Densely Inhabited Districts (DIDs), in Sendai City, Miyagi Prefecture, Japan (Fig. 1). Sendai City is the regional capital in the Northeast region of Japan and consists of five administrative districts: Aoba-ku, Izumi-ku, Miyagino-ku, Taihaku-ku, and Wakabayashi-ku. According to the 2020 Population Census, it has a total population of 1,096,704. Since the period of high economic growth in the 1960s, the residential areas initially developed on plateau areas have spread to hilly suburbs [27]. The city centre of Sendai is surrounded by nature, such as the Hirose River and the Aobayama hills, and the area is adorned with abundant greenery, including tree-lined streets [28].

### Participants

This study used self-reported questionnaires from the baseline survey of the Tohoku Medical Megabank Community-Based Cohort Study (﻿TMM CommCohort Study). The TMM CommCohort Study recruited ﻿people aged 20 years old and over, who were registered in the basic resident register of all municipalities in Miyagi and Iwate Prefectures at the time of enrolment. The survey was ﻿performed at specific municipal health check sites, the Community Support Centre in ﻿Tohoku Medical Megabank Organization (ToMMo), and the satellite in Iwate Medical University Iwate Tohoku Medical Megabank Organization (IMM) [29]. The baseline survey was conducted between 2013 and 2015. Of the 17,688 participants in the TMM CommCohort baseline survey who took the type 2 survey (including those who participated in the type 2 survey after the type 1 survey), 16,928 consented and participated as of 19 August 2021. Of those, 4450 participants who resided in the DIDs in Sendai City were included in the analysis. The human subjects’ committee of the Tohoku Medical Megabank Organization Institution, Tohoku University approved the survey protocol (approval no. 2019-4-065 and 2019-4-032). Informed consent was obtained from all participants, including assuring voluntary participation and the right to withdraw at any time. The analysis was designed and conducted in accordance with the applicable guidelines and regulations for the use and analysis of the TMM CommCohort Study.

### Outcome

Leisure walking time per week (min), based on the validated self-reported questionnaires of the TMM CommCohort Study, was used as an indicator of walking behaviour [30]. In the questionnaires, the participants answered the frequency and average duration of leisure walking, by which the leisure walking time per week was calculated for each participant according to the following formula [31]: leisure walking time (min/week) = duration (h) × 60 (min/h) × frequency (frequency/day) × 7 (day/week). Participants provided an average frequency over the course of one year to eliminate seasonal differences. The average duration categories (assigned average hours per activity) were: < 30 min (0.25), 30 min to 1 h (0.75), 1 to < 2 h (1.5), 2 to < 3 h (2.5), 3 to < 4 h (3.5), and ≥ 4 h (4.0). The frequency categories (assigned average frequency per day) for leisure walking in the questionnaire were: almost none (0), less than once per month (0.5/30), one to three times per month (2/30), one to two times per week (1.5/7), three to four times per week (3.5/7 = 0.5), and almost every day (7/7 = 1.0).

### Streetscape indices of greenery

A greenery index was developed using GSV images. We used green visibility, which considers height from the viewer’s perspective. This facilitated a visual understanding of how the overhead greenery are perceived by a pedestrian on the street.

(1) Obtaining GSV images

ArcMap 10.8.1 (ESRI Inc.) was used to set up sample sites for streetscape assessment at 50-m intervals on roads in DIDs and within 1 km from the edge of DIDs in Sendai City. The road data were obtained from ArcGIS GeoSuite Road Network (Esri Japan Inc.) as of 2020. Points on motorways (Sendai Nishi Road, Sendai-Tobu Road, Sendai-Nanbu Road, Tohoku Expressway, and Sanriku Expressway) were excluded. The final set contained 65,762 landscape assessment observations. We obtained GSV images for each sample site using the following procedure. First, metadata were obtained for the closest GSV images taken within a 10 m-radius of each sample site in each year from 2013 to 2015. The metadata include PanoID, latitude, longitude, year and month of image acquisition, and direction of the image. PanoID is a unique ID assigned to each GSV image. It consists of numbers, letters, and symbols, with a total of 22 characters. We could obtain the metadata for GSV images of 59,429 (90.64%) of the sites. The metadata for GSV images of 6333 (9.36%) of the sites could not be acquired and were considered missing values. Second, GSV images (1664 px × 832 px) were obtained based on PanoID. To account for seasonality, images taken during the greener months of April to September were preferentially targeted. For sample sites where only images taken in January to March or October to December were available, these GSV images were used. Of the 59,429 images, 1121 (1.89%) were taken in spring (March to May), 39,895 (67.13%) in summer (June to August), 18,412 (30.98%) in autumn (September to November), and 1 (0.00%) in winter (January, February and December).

(2) Green extraction

A machine learning approach was implemented to identify green areas from the downloaded GSV images. We used DeepLab v3 + [32] to perform the semantic segmentation of images. DeepLab v3 + is a deep learning model for semantic segmentation. Semantic segmentation is a method of classifying images into ﻿pixel-wise labels of semantic classes of objects.

Following Nagata et al. [15], this study used a pre-trained model of the Cityscapes Dataset [33] as training data and classified each pixel of the GSV image into 19 classes. Each classified pixel was assigned a corresponding label. The Cityscapes Dataset has 30 classes defined for annotations. Those classes are based on eight groups: ﻿flat, construction, nature, vehicle, sky, object, human, and void. In the dataset, classes that are too rare are excluded, leaving 19 classes for evaluation [33]. In this study, as per Xia et al. [34], pixels classified as vegetation classes were treated as green.

(3) Calculation of green visibility

**Fig. 2:**
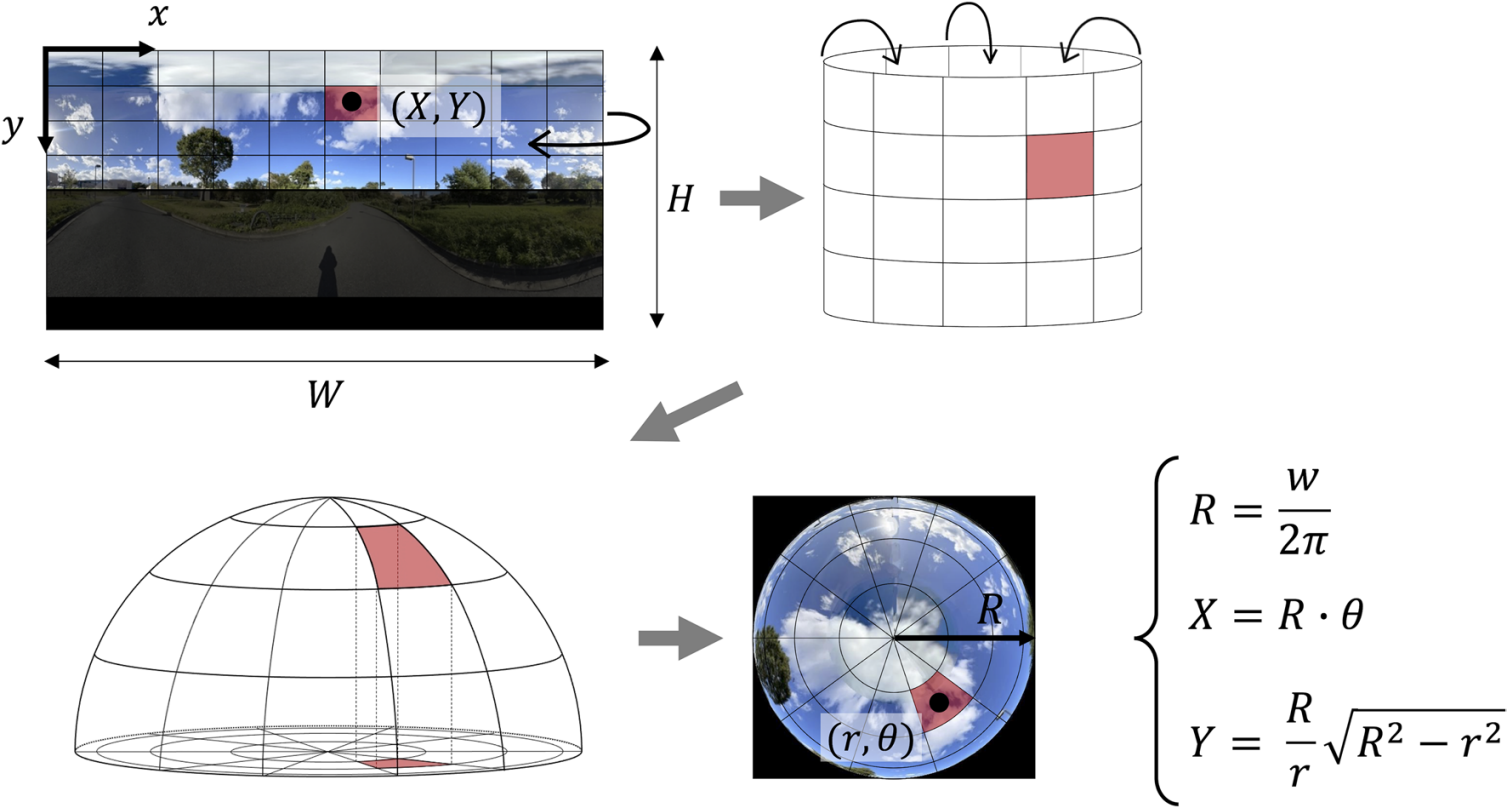
Transformation of the cylindrical GSV panorama to fisheye image

GSV images acquired using equi-cylindrical projection were transformed into a sky map (orthographic projection method) following the method presented by Nishio and Ito [35] (Fig. 2). Green visibility was calculated as follows.

Given that *W* is the width of the GSV image, $(\mathrm{X},\mathrm{ Y})$ are the orthogonal coordinates of a point in the GSV image, the corresponding point in the sky map in polar coordinates $(\mathrm{r},\uptheta )$, and *R* is the radius of the sky chart, the following relationship holds:

$$R\, = \,\frac{W}{2\pi },\,X\, = \,R \cdot \theta ,\,Y = \frac{R}{r} \cdot \sqrt {R^{2} - r^{2} }$$

We defined overall green visibility as the ratio of the number of pixels classified as vegetation class to the number of pixels in the whole sky part of the sky map obtained using the above equation.

**Fig. 3:**
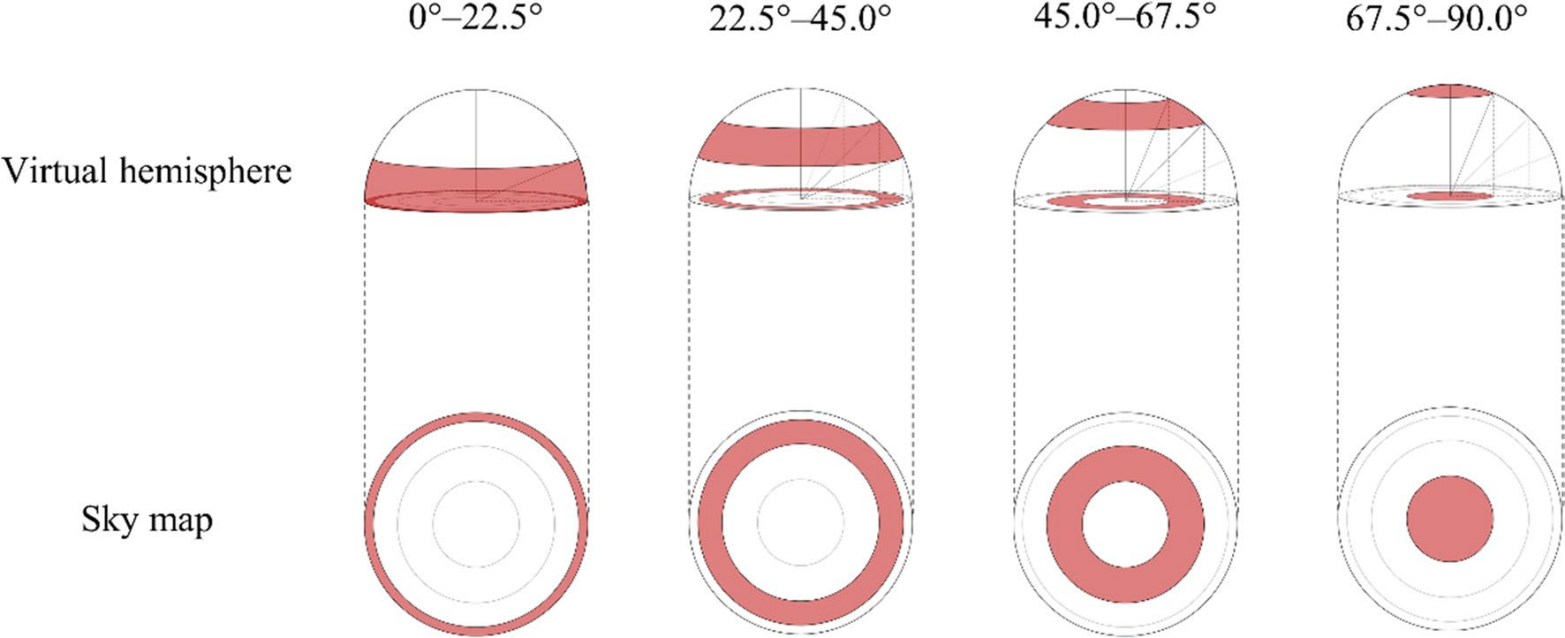
Green visibility separated by latitude

The sky map was classified into four ranges (0°–22.5°; 22.5°–45.0°; 45.0°–67.5°; 67.5°–90.0°) according to the latitude of the virtual hemisphere, and the ratio of the number of pixels classified as vegetation class to those in all parts of each range was calculated (green visibility separated by latitude) (Fig. 3). Higher latitude range indices represent higher green visibility from the viewpoint location (90° corresponded to zenith).

(4) Neighbourhood indicators of street greenery

Following previous studies [36, 37], we defined the neighbourhood of a participant as the area within the road network buffer within 1000 m from their residence. The mean green visibility on the streets within this area was computed for each participant. This study included only participants with at least 20 sampling sites where the GSV image could be obtained in the buffer; thus, 4431 participants were included and 19 excluded.

### Statistical analysis

We conducted multilevel regression analysis (mixed-effect models) in R 4.1.0 using the ‘lme4’ package [38] to investigate the relationships between leisure walking behaviour and the streetscape indicators of green in the neighbourhood of a participant. We set the leisure walking hours per week as the dependent variable, and each of five neighbourhood indicators of green (green visibility of the whole range or four green visibility zones separated by latitude) as the independent variable in the regression analysis. To ensure result robustness, the models were used with a random intercept at the elementary school district level to account for possible clustering tendency of walking hours due to unknown neighbourhood factors.

Model A only considers the age group (20 s; 30 s; 40 s; 50 s; 60 s; $\ge$ 70) and gender (male; female) as control variables. In Model B, we added the following variables as control variables to Model A: marital status (married; separated; widowed; single), education (primary or junior high school; high school; junior college, technical college or vocational school; university or graduate school; other), family size (number of members, including self), employment status (employed with income; unemployed), alcohol consumption (never; unable; current; former), degree of urbanization (calculated based on the population density of the neighbourhood), and the number of urban parks in a neighbourhood (as an indicator of recreational neighbourhood resource). Population data were obtained from the 2015 Population Census using the quarter grid square data (Statistics Bureau, 1996), which counts population for each grid cell of approximately 250 m side-length. We calculated population density by distributing the population in each grid proportionally by the area where it intersects the 1000-m network buffer from the residence and dividing by the area of the buffer. We included alcohol consumption as a control variable of health awareness. The data concerning urban parks was obtained from the digital national land information provided by the Ministry of Land, Infrastructure, Transport, and Tourism of Japan (https://nlftp.mlit.go.jp/ksj/gml/datalist/KsjTmplt-P13.html).

In a series of regression analyses, we conducted a complete-case analysis.

## Results

### Participant characteristics

A total of 4280 participants were included in the analysis (1241 men, 3039 women) excluding 151 participants with missing variables. Table 1 illustrates the summary statistics of the participants. Most participants (n = 3294, 79.96%) were married. The number of employed and unemployed participants was roughly the same (approximately 2100). The largest age group was 60–69, with 1479 (34.6%). In terms of streetscape indicators, the mean green visibility of the whole area was 6.98% and that by latitudinal zone was 37.82% for 0.0°–22.5°, 22.05% for 22.5°–45.0°, 10.94% for 45.0°–67.5°, and 5.54% for 67.5°–90.0°. The mean leisure walking time per week was 15.24 min.

**Table 1:** Participant characteristics

	n, mean	( %, SD)
Age		
20–29	150	(3.5)
30–39	392	(9.2)
40–49	655	(15.3)
50–59	757	(17.7)
60–69	1479	(34.6)
70-	847	(19.8)
Gender		
Male	1241	( 29.0)
Female	3039	(71.0)
Martial status		
Married	3294	(77.0)
Divorced or widowed	421	(9.8)
Single	565	(13.2)
Education		
Primary school or junior high school	145	(3.4)
Senior high school	1852	(43.3)
Junior college, technical school or vocational school	1161	(27.1)
College or above	1101	(25.7)
Others	21	(0.5)
Family size		
1	485	(11.3)
2	1710	(40.0)
3	983	(23.0)
4	688	(16.1)
5	250	(5.8)
6 and more	164	(3.8)
Working status		
Working with income	2175	(50.8)
Not working	2105	(49.2)
Alcohol consumption		
Never	2540	(59.3)
Unable	89	(2.1)
Current	1408	(32.9)
Former	243	(5.7)
Residential density (/km^2^)		
≦4000	652	(15.2)
4001–6000	1428	(33.4)
6001–8000	862	(20.1)
8001–10000	683	(16.0)
10,001–12,000	389	(9.1)
> 12,000	266	(6.2)
Number of urban parks	11.75	(7.2)
Overall green visibility (%)	6.98	(3.5)
Green visibility separated by latitude (%)		
Green visibility 0.0°–22.5°	37.82	(12.8)
Green visibility 22.5°–45.0°	22.05	(8.5)
Green visibility 45.0°–67.5°	10.94	(4.7)
Green visibility 67.5°–90.0°	5.54	(3.0)
Leisure walking (min/week)	104.75	(191.8)

### Spatial distribution of green visibility

**Fig. 4:**
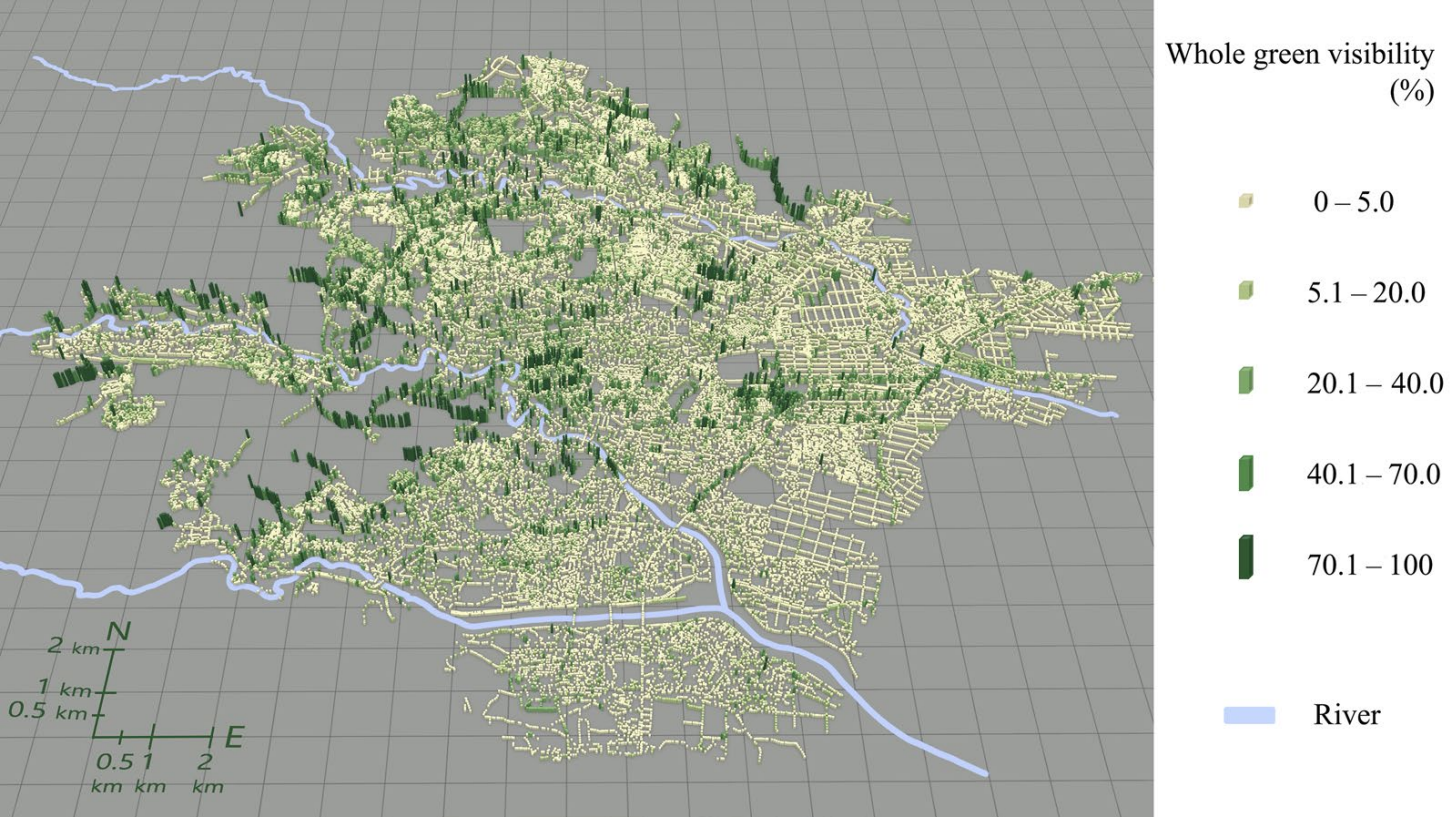
Spatial distribution of overall green visibility at observation points along streets

Figure 4 illustrates the spatial distribution of the overall green visibility in the DIDs in Sendai City. When comparing the southern and northern areas, it becomes evident that the northern area tends to exhibit greater overall green visibility. Additionally, there exists a continuous alignment of points along a specific street, exhibiting notably high values for green visibility, particularly along the tree-lined streets in the city centre. Overall green visibility is also higher in the areas surrounding the city centre where greenery is present.

**Fig. 5:**
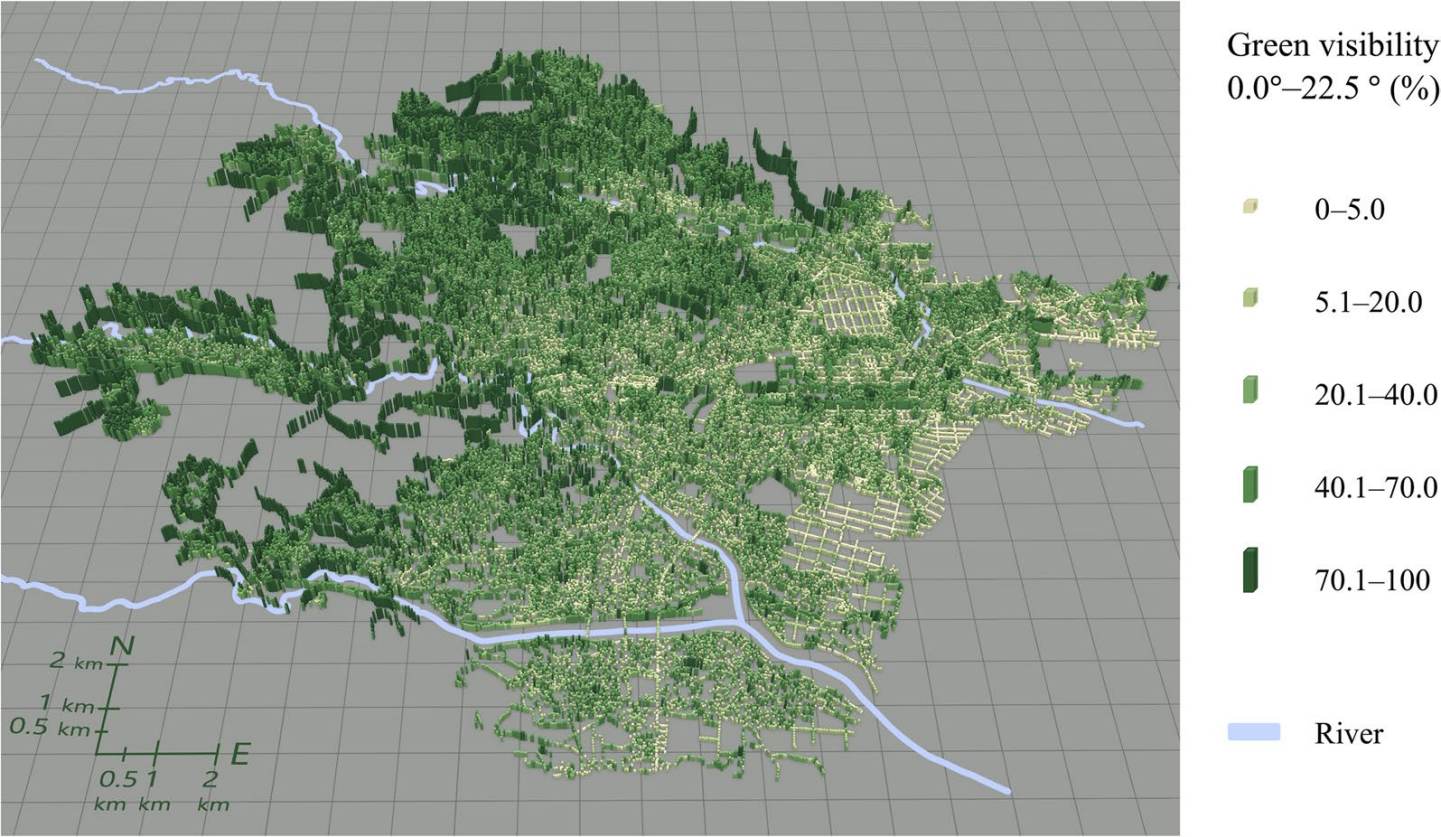
Spatial distribution of green visibility 0.0°–22.5° at observation points along streets

**Fig. 6:**
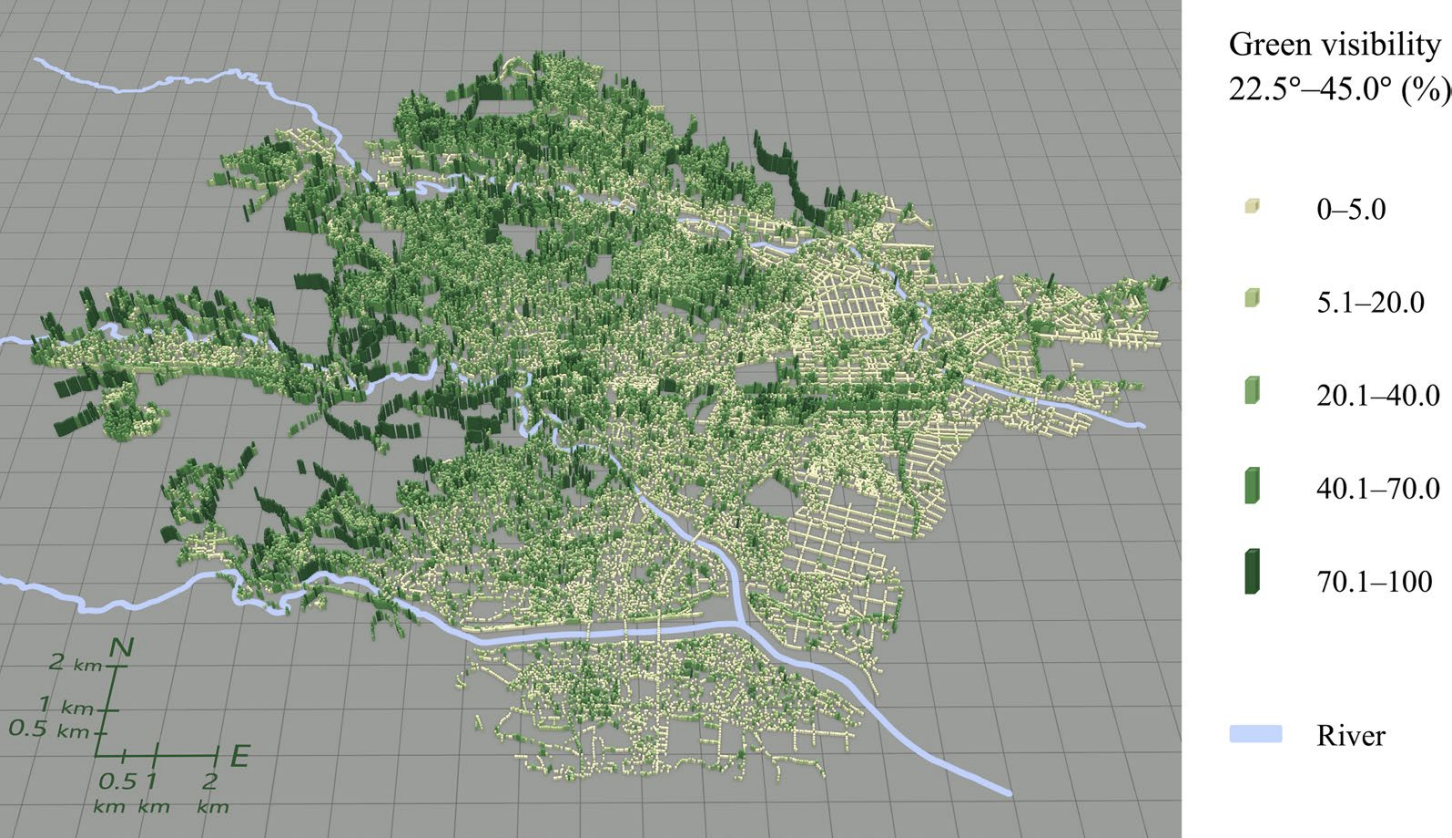
Spatial distribution of green visibility 22.5°–45.0° at observation points along streets

**Fig. 7:**
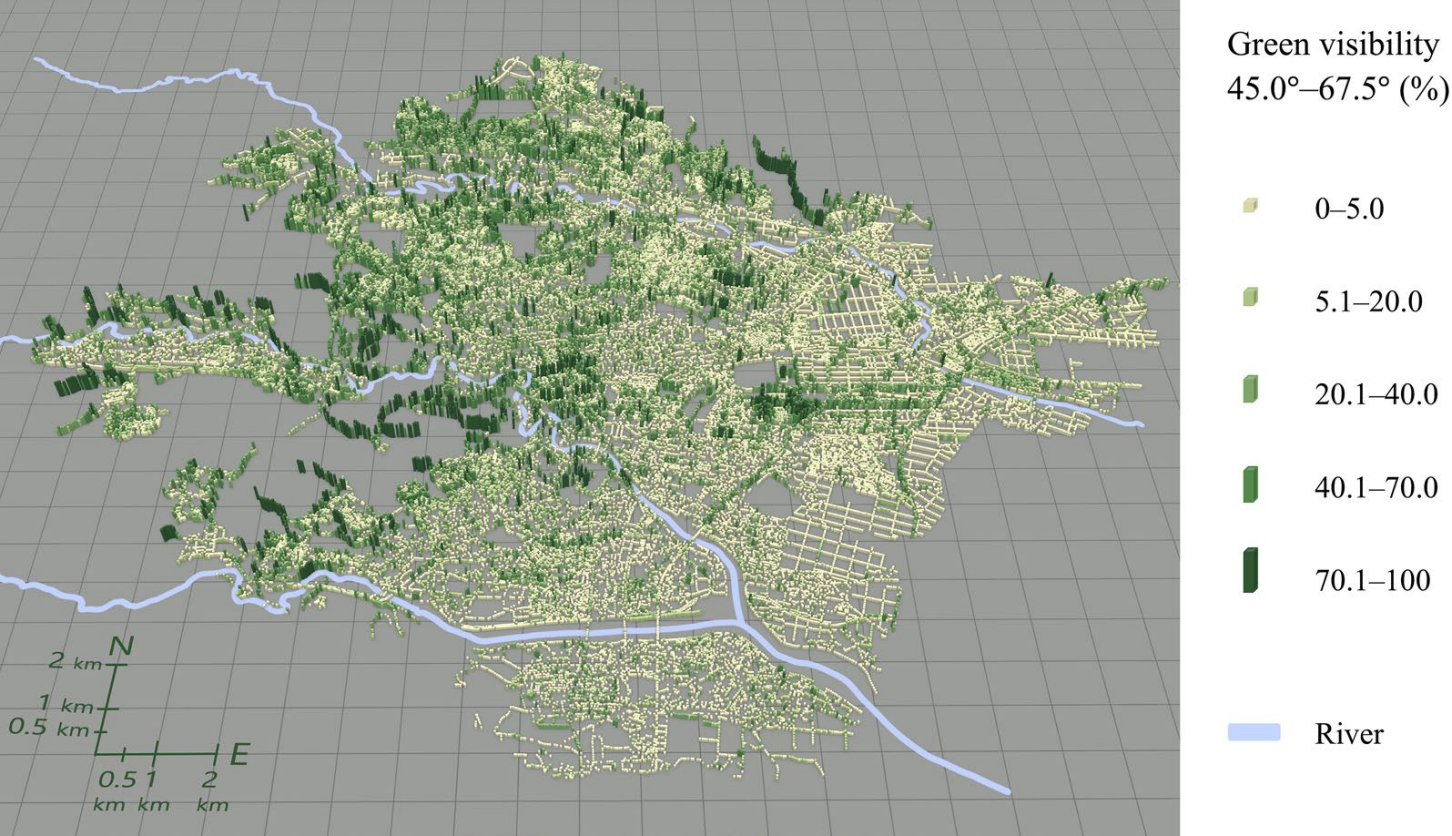
Spatial distribution of green visibility 45.0°–67.5° at observation points along streets

**Fig. 8:**
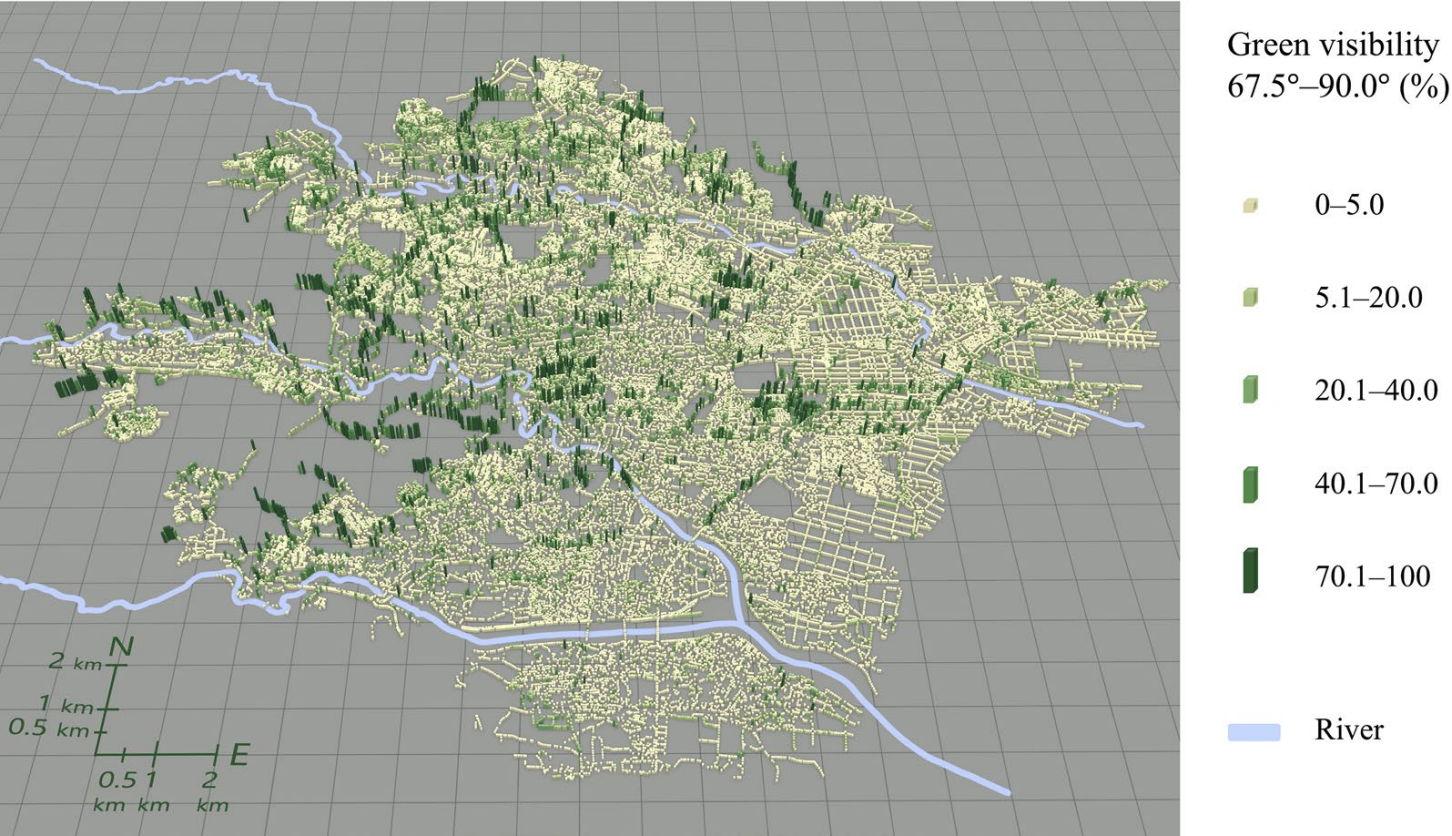
Spatial distribution of green visibility 67.5°–90.0° at observation points along streets

Figures 5, 6, 7, 8 illustrate the spatial distribution of each green visibility separated by latitude in the DIDs in the city. For green visibility in the lower latitude parts of the virtual hemisphere (0°–22.5°, 22.5°–45.0°), the points with higher values are more widely distributed. On the other hand, for green visibility in the higher latitude parts, high green visibility points are concentrated on the streets in the middle of the diagram, such as Jozenji Street and Aoba Street, which are well-known for being tree-lined avenues. For green visibility in the lower latitude parts, as in overall green visibility, values tend to be higher in the north of the study area and lower in the south.

### Walking and neighbourhood green visibility

Table 2 indicates the results of the statistical analyses for the association between leisure walking and neighbourhood green visibility. In Model A, there was a statistically significant positive association between leisure walking time per week and overall green visibility. For green visibility separated by latitude, no significant associations were found between leisure walking time per week and 0°–22.5° or 22.5°–45.0°. However, there were statistically significant associations between walking hours per week and 45°–67.5°, as well as 67.5°–90°.

**Table 2:** Associations between green visibility and leisure walking based on multilevel models

	Model a			Model b		
	β	95% CI	AIC	β	95% CI	AIC
Overall green visibility	**2.534**	**0.675–4.397**	5355.4	**1.938**	**0.077–3.793**	5386.5
Green visibility 0.0°–22.5°	− 0.235	− 0.760–0.291	5364.3	− 0.222	− 0.780–0.338	5401.4
Green visibility 22.5°–45.0°	0.257	− 0.528–1.044	5363.8	0.184	− 0.605–0.973	5392.2
Green visibility 45.0°–67.5°	**1.588**	**0.210–2.969**	5358.0	1.212	− 0.165–2.585	5397.2
Green visibility 67.5°–90.0°	**3.065**	**0.933–5.199**	5354.4	**2.385**	**0.247–4.514**	5385.7

Results are presented as β [95% confidence interval (CI)] per 1 point increase

**Bold**: significant at 5% level

Model A: adjusted for age and gender

Model B: Model A + marital status, education, family size, working status, alcohol consumption, population density and number of urban parks

In Model B, there was a statistically significant positive association between leisure walking time per week and overall green visibility. For green visibility separated by latitude, there was no significant association between leisure walking duration and 0°–22.5°, 22.5°–45.0° or 45°–67.5°. There was a statistically significant positive association between leisure walking time and 67.5°–90°.

Note that the estimated coefficients of green visibility were greater with higher latitude zones. In all analyses of the association between green visibility and leisure walking, the results of Models A and B were consistent. Models focusing on high latitude greens (67.5°–90°) have better AIC (lower AIC). Although the AIC differences between the models for overall green visibility and the highest latitude green visibility were low, it is clear that the models for high latitude green visibility performed statistically better than the ones for lower latitude green visibility.

## Discussion

The models we applied demonstrated that people living in neighbourhoods with greater overall green visibility spent more time per week leisure walking. This suggests that an increased presence of greenery on neighbourhood streets correlated with an extended walking time among residents, even after controlling for individual socioeconomic status and health awareness, such as alcohol consumption. This is consistent with previous studies [7–10] that have reported a positive association between the amount of greenery in neighbourhood streets and physical activity, particularly walking behaviour. Moreover, the study revealed that green visibility specifically at higher latitudes (67.5°–90°) within the pedestrian's streetscape significantly correlated with the leisure walking time per week in the models. It should be highlighted that the coefficient was greater for higher latitude zones, which indicates that the amount of greenery at higher positions in the visual field was strongly associated with leisure walking time.

There are two distinct yet interconnected explanations regarding how the vertical dimension of greenery impacts leisure walking: namely, the potential physical and psychological influences stemming from the presence of greenery at elevated positions, which collectively contribute to a more favourable walking experience on the streets.

With respect to the physical implications, it has been observed that the shadows cast by tall trees foster an ideal setting for outdoor physical activities and walking, providing a balance of moderate light and thermal comfort while minimizing UV exposure [24]. Lee et al. [39] reported that pedestrians prefer to walk in shaded areas due to the thermal comfort it provides, particularly during the summer months. Villeneuve et al. [40] using a survey on participation in recreational physical activities by season reported the streetscape greenery index was positively associated with recreational activities during the summer. As this study examined average year-round levels of leisure walking time, it was not possible to investigate the effects of seasonal variation in detail. Considering that physical activity, including walking, is typically more prevalent during non-winter months compared to winter months [41, 42], our analysis likely captured the association between leisure walking activity in non-winter months and the streetscape during that period. It should be noted that the high latitude greens considered in this study pertain to those situated above the pedestrian's viewpoint in the hypothetical hemisphere. Thus, they directly account for the green shadows that pedestrians encounter on the street. Furthermore, tall greenery can have indirect physical implications, including noise reduction and mitigation of environmental pollution, which pedestrians may perceive [23].

Regarding the psychological aspects, several past studies indicate that greenery higher than the eye-level height offers an aesthetically favourable landscape promoting walking with people's mental comfort or a sense of “refuge” as introduced in the introductory section [17, 22, 26]. The aesthetics of streetscapes are well known to be positively correlated with leisure walking [5], and shade created by greenery is also considered to be a component of an aesthetic landscape [43, 44]. Moreover, Hu et al. [22] have demonstrated that a small gap between tree canopies and a high number of peaks in the tree canopy line are positively correlated with perceptions of favourable streetscape aesthetics. This means that a dense arrangement of tall trees is more likely to be rated as a beautiful streetscape. Therefore, the greater the green visibility at higher positions as viewed by a pedestrian, the more likely the streetscape is to be rated as beautiful, thus encouraging walking.

This study has several strengths. We used GSV, which contributes to the development of an objective method for assessing the amount of greenery divided by height. Previous methods have been limited by the fact that the amount of greenery was measured using indicators such as parks, number of trees, and normalized difference vegetation index, which may differ from the number of green spaces perceived by people, especially in areas with dense vegetation [12, 19]. Second, this study investigated which height of greenery is associated with leisure walking. Previous studies that only investigated the relationship between greenery in the streetscape and walking analysed the overall amount of greenery in the streetscape. By contrast, this study has demonstrated that greenery at higher positions is associated more with walking than greenery at lower positions. This indicates that an increased amount of greenery at higher positions may contribute to higher quality greenery, thus promoting leisure walking. By focusing on the height of street trees, this study has taken a further step compared to previous research that examined the relationship between the overall presence of street greenery and leisure walking. Further, it is expected that not only the amounts of segmented objects, such as greenery in a streetscape, but also their spatial arrangement may determine the quality of the streetscape that promote leisure walking.

Nevertheless, this study is not without limitations. First, it did not analyse the causal relationship between greenery and leisure walking. In addition to the possibility that tree shade promotes leisure walking, it is also possible that people who engage in leisure walking regularly prefer areas with a significant amount of tree shade and choose these areas as their residence. Further analyses, such as a longitudinal analysis, may be needed to explore the self-selection effect. Second, dense vegetation that creates blind spots could make pedestrians perceive the environment as unsafe [45]; it has been reported that perceived sidewalk safety is related to walking behaviour [46]. As this study cannot determine whether vegetation creates blind spots that cause crime risk, it may be necessary to consider new ways of assessing the walking environment from the perspective of safety in the future studies. Third, there should be seasonal variations in the effects of the height of street greenery. Han et al. [47] demonstrated a method to discern seasonal disparities in street greenery using streetscape images. Further research is required to clarify the seasonal and temporal variations by employing a series of streetscape images from different periods.

## Conclusions

This study investigated how greenery in the streetscape is associated with leisure walking. The results revealed that greenery positioned at higher levels is associated more closely with leisure walking time. A possible reason for this is that greenery in higher positions contributes to thermally comfortable and aesthetic streetscapes, thus promoting leisure walking. Moreover, the indices that can contribute to a desirable neighbourhood environment were found to be spatially uneven in the areas covered in this study. For neighbourhoods with low levels of physical activity, increasing the amount of greenery particularly at higher positions, may encourage residents to increase their leisure walking time, leading to a reduction of geographical disparities in health.

## Data Availability

The personal data, including residential information and leisure walking time, are not publicly accessible due to privacy settings outlined in the project contract. However, other materials may be made available by contacting the corresponding author upon reasonable request.

## Abbreviations

GSV: Google Street View
DIDs: Densely Inhabited Districts
LiDAR: Light Detection and Ranging
TMM CommCohort Study: Tohoku Medical Megabank Community-Based Cohort Study
ToMMo: Tohoku Medical Megabank Organization
IMM: Iwate Tohoku Medical Megabank Organization

## References

[1] Kekäläinen T, Freund AM, Sipilä S, Kokko K (2020). Cross-sectional and longitudinal associations between leisure time physical activity, mental well-being and subjective health in middle adulthood. Appl Res Qual Life.

[2] Kim J, Lee J, Kim Y-S, Park S-H (2022). Identifying the relationship between leisure walking and prevalence of Alzheimer’s disease and other dementias. Int J Environ Res Public Health.

[3] Prince SA, Lancione S, Lang JJ, Amankwah N, de Groh M, Jaramillo Garcia A (2022). Examining the state, quality and strength of the evidence in the research on built environments and physical activity among adults: an overview of reviews from high income countries. Health Place.

[4] Owen N, Humpel N, Leslie E, Bauman A, Sallis JF (2004). Understanding environmental influences on walking; Review and research agenda. Am J Prev Med.

[5] Sugiyama T, Cerin E, Owen N, Oyeyemi AL, Conway TL, Van Dyck D (2014). Perceived neighbourhood environmental attributes associated with adults׳ recreational walking: IPEN Adult study in 12 countries. Health Place.

[6] Inoue S, Ohya Y, Odagiri Y, Takamiya T, Ishii K, Kitabayashi M (2010). Association between perceived neighborhood environment and walking among adults in 4 Cities in Japan. J Epidemiol.

[7] Yang Y, He D, Gou Z, Wang R, Liu Y, Lu Y (2019). Association between street greenery and walking behavior in older adults in Hong Kong. Sustain Cities Soc.

[8] Lu Y, Sarkar C, Xiao Y (2018). The effect of street-level greenery on walking behavior: evidence from Hong Kong. Soc Sci Med.

[9] Ki D, Lee S (2021). Analyzing the effects of green view index of neighborhood streets on walking time using google street view and deep learning. Landsc Urban Plan.

[10] Lu Y (2019). Using Google Street View to investigate the association between street greenery and physical activity. Landsc Urban Plan.

[11] Ewing R (1996). Pedestrian-and Transit-Friendly Design.

[12] Li X, Zhang C, Li W, Ricard R, Meng Q, Zhang W (2015). Assessing street-level urban greenery using Google Street View and a modified green view index. Urban For Urban Green.

[13] Rzotkiewicz A, Pearson AL, Dougherty BV, Shortridge A, Wilson N (2018). Systematic review of the use of Google Street View in health research: major themes, strengths, weaknesses and possibilities for future research. Health Place.

[14] Biljecki F, Ito K (2021). Street view imagery in urban analytics and GIS: a review. Landsc Urban Plan.

[15] Nagata S, Nakaya T, Hanibuchi T, Amagasa S, Kikuchi H, Inoue S (2020). Objective scoring of streetscape walkability related to leisure walking: statistical modeling approach with semantic segmentation of google street view images. Health Place.

[16] Aikoh T, Homma R, Abe Y (2023). Comparing conventional manual measurement of the green view index with modern automatic methods using google street view and semantic segmentation. Urban For Urban Green.

[17] Ulmer JM, Wolf KL, Backman DR, Tretheway RL, Blain CJ, O’Neil-Dunne JP (2016). Multiple health benefits of urban tree canopy: the mounting evidence for a green prescription. Health Place.

[18] Tsai W-L, Yngve L, Zhou Y, Beyer KMM, Bersch A, Malecki KM (2019). Street-level neighborhood greenery linked to active transportation: A case study in Milwaukee and Green Bay. WI, USA Landsc Urban Plan.

[19] Jiang B, Deal B, Pan H, Larsen L, Hsieh C-H, Chang C-Y (2017). Remotely-sensed imagery vs. eye-level photography: evaluating associations among measurements of tree cover density. Landsc Urban Plan.

[20] van Dillen SME, de Vries S, Groenewegen PP, Spreeuwenberg P (1978). Greenspace in urban neighbourhoods and residents’ health: adding quality to quantity. J Epidemiol Community Health.

[21] de Vries S, van Dillen SME, Groenewegen PP, Spreeuwenberg P (2013). Streetscape greenery and health: Stress, social cohesion and physical activity as mediators. Soc Sci Med.

[22] Hu T, Wei D, Su Y, Wang X, Zhang J, Sun X (2022). Quantifying the shape of urban street trees and evaluating its influence on their aesthetic functions based on mobile lidar data. ISPRS J Photogramm Remote Sens.

[23] Reid C, Clougherty J, Shmool J, Kubzansky L (2017). Is all urban green space the same? A comparison of the health benefits of trees and grass in New York City. Int J Environ Res Public Health.

[24] Tabatabaie S, Litt JS, Carrico A (2019). A study of perceived nature, shade and trees and self-reported physical activity in Denver. Int J Environ Res Public Health.

[25] Appleton J (1996). The experience of landscape.

[26] Cao Y, Huang L (2023). A study on the impact of small-scale courtyard landscape layouts on spatial oppressiveness in dense high-rise environments. Sustainability.

[27] Chiba A (2006). The development process and regional problems of large-scale residential areas in the sendai metropolitan area after 1991. Urban Geogr.

[28] Sendai City Government (2021). Sendai city green master plan 2021–2030.

[29] Hozawa A, Tanno K, Nakaya N, Nakamura T, Tsuchiya N, Hirata T (2021). Study profile of the tohoku medical megabank community-based cohort study. J Epidemiol.

[30] Fujii H, Yamamoto S, Takeda-Imai F, Inoue M, Tsugane S, Kadowaki T (2011). Validity and applicability of a simple questionnaire for the estimation of total and domain-specific physical activity. Diabetol Int.

[31] Nakaya N, Xie T, Scheerder B, Tsuchiya N, Narita A, Nakamura T (2021). Spousal similarities in cardiometabolic risk factors: a cross-sectional comparison between dutch and japanese data from two large biobank studies. Atherosclerosis [Internet].

[32] Chen L-C, Zhu Y, Papandreou G, Schroff F, Adam H, Ferrari V, Hebert M, Sminchisescu C, Weiss Y (2018). Encoder-decoder with atrous separable convolution for semantic image segmentation. Computer Vision—ECCV 2018.

[33] Cordts M, Omran M, Ramos S, Rehfeld T, Enzweiler M, Benenson R (2016). The cityscapes dataset for semantic urban scene understanding. IEEE Conf Comput Vis Pattern Recogn.

[34] Xia Y, Yabuki N, Fukuda T (2021). Development of a system for assessing the quality of urban street-level greenery using street view images and deep learning. Urban For Urban Green.

[35] Nishio S, Ito F (2019). Application of method for calculating sky view factor using google street view: relation between sky view factor and physical elements in urban space. Proc ICA.

[36] Frank LD, Andresen MA, Schmid TL (2004). Obesity relationships with community design, physical activity, and time spent in cars. Am J Prev Med.

[37] Carlson JA, Saelens BE, Kerr J, Schipperijn J, Conway TL, Frank LD (2015). Association between neighborhood walkability and GPS-measured walking, bicycling and vehicle time in adolescents. Health Place.

[38] Bates D, Mächler M, Bolker B, Walker S (2014). Fitting Linear Mixed-Effects Models using lme4. arXiv.

[39] Lee LSH, Cheung PK, Fung CKW, Jim CY (2020). Improving street walkability: Biometeorological assessment of artificial-partial shade structures in summer sunny conditions. Int J Biometeorol.

[40] Villeneuve PJ, Ysseldyk RL, Root A, Ambrose S, DiMuzio J, Kumar N (2018). Comparing the normalized difference vegetation index with the google street view measure of vegetation to assess associations between greenness, walkability, recreational physical activity, and health in Ottawa, Canada. Int J Environ Res Public Health.

[41] Sun Y, Wang X, Zhu J, Chen L, Jia Y, Lawrence JM (2021). Using machine learning to examine street green space types at a high spatial resolution: application in Los Angeles County on socioeconomic disparities in exposure. Sci Total Environ.

[42] Hamilton SL, Clemes SA, Griffiths PL (2008). UK adults exhibit higher step counts in summer compared to winter months. Ann Hum Biol.

[43] Handy SL, Boarnet MG, Ewing R, Killingsworth RE (2002). How the built environment affects physical activity. Am J Prev Med.

[44] Saelens BE, Sallis JF, Black JB, Chen D (2003). Neighborhood-based differences in physical activity: an environment scale evaluation. Am J Public Health.

[45] Jansson M, Fors H, Lindgren T, Wiström B (2013). Perceived personal safety in relation to urban woodland vegetation—a review. Urban For Urban Green.

[46] Hong J, Chen C (2014). The role of the built environment on perceived safety from crime and walking: examining direct and indirect impacts. Transportation.

[47] Han Y, Zhong T, Yeh AGO, Zhong X, Chen M, Lü G (2023). Mapping seasonal changes of street greenery using multi-temporal street-view images. Sustain Cities Soc.

